# Evaluation of ‘For survivors by survivors’, a cancer survivorship peer support pilot program for healthcare staff: A one-group post-test-only study

**DOI:** 10.1371/journal.pone.0337591

**Published:** 2025-12-02

**Authors:** Ciaran Evans, Maria O’Malley, Mark R. O’Donovan, Irem Koc, Caroline Kilty, Sean Kelleher, Teresa Wills, Josephine Hegarty, Mohamad M. Saab

**Affiliations:** 1 School of Nursing and Midwifery, University College Cork, Cork, Ireland; 2 School of Medicine, University College Cork, Cork, Ireland; Touro University California College of Pharmacy, UNITED STATES OF AMERICA

## Abstract

**Introduction:**

The number of people living with and beyond cancer has risen significantly due to medical advancements. The cancer journey remains physically, socially, and psychologically challenging. Peer support is important in cancer survivorship as it provides emotional validation and understanding, practical advice and a sense of belonging. A peer support program was developed to assist healthcare staff in Ireland who have had a diagnosis of cancer in their recovery and engagement with their workplace and work.

**Aim:**

To examine and evaluate the effectiveness and impact of the “for survivors by survivors” pilot cancer survivorship program for the peer supporter, the person being supported (peer) and the line manager of the peer supporter.

**Methods:**

Three researcher-designed surveys were distributed via a voluntary cancer awareness and support services organization that acted as the gatekeeper. Data were collected on demographic and program-related factors and experience. Data were then analyzed using descriptive and inferential statistics, with open-ended questions analyzed using inductive content analysis.

**Findings:**

For all three groups (peers, peer supporters and line managers; n = 42) 98% would recommend the program in the future. For peers 74% (n = 11) reported increased emotional well-being after interactions with their peer supporter; and large statistically significant improvements for communication, knowledge, information-seeking and self-confidence (effect sizes r = 0.67–0.80; p-values<0.05). For peer supporters, 84% (n = 16) evaluated the training and program positively, and 42% (n = 8) preferred in-person training. For line managers 88% (n = 7) were satisfied with the program. The most common barrier to participation for all three groups was time constraints. Awareness of the program was reported as low.

**Conclusion:**

Peer support programs for cancer survivors can enhance emotional well-being at work. The effectiveness of such programs is constrained by practical barriers such as time limitations and low program awareness. Addressing these issues is essential for improving program reach and impact.

## 1. Introduction

Cancer survivorship describes a person living with and beyond cancer [1]. Globally, nearly 20 million new cancer incidences were reported by the World Health Organization in 2022, with 53.5 million people alive within five years of a cancer diagnosis [2]. There were 220,728 cancer survivors in Ireland at the end of 2022, about 4.3% of the population [3] The number of people living with and beyond cancer in Ireland has increased by more than 50% in the last decade with this number expected to double over the next 25 years. This is attributed to advancements in detection, treatment, and supportive care [1]. Diagnosis, treatment and follow up care can be a traumatic experience that often has physical, social and psychological implications for cancer survivors [4].

Addressing the informational, emotional and psychological needs of cancer survivors is strongly recommended [5]. In alignment with this, Ireland's 2017–2026 National Cancer Strategy places significant emphasis on survivorship, underscoring the importance of high-quality care to support recovery and adjustment to the range of chronic physical, psychological and social issues experienced after cancer [1]. Peer support may cultivate hope, help with fear of cancer recurrence or progression, and alleviate anxiety [4], as well as encourage confidence in cancer survivors [6]. Several peer support programs have been developed to provide reassurance to those diagnosed with cancer. One such novel pilot cancer survivorship peer support service, “for survivors by survivors” was developed in Ireland in 2023 in partnership with the Marie Keating Foundation (a voluntary cancer awareness and support services organization). This program was devised as a service for health service employees to assist in their recovery and engagement with their workplace either during diagnosis, treatment, returning to work and managing any challenges in the workplace [7]. Thirty health service employees with a personal history of cancer were initially trained to act as peer supporters. This training featured three in-person training sessions encompassing peer supporter role and responsibilities, including peer supporter self-care, role-play exercises (listening skills, negotiating support agreements) and speakers with lived experience of peer support. This was supplemented with a follow-up continuing practice development session which was delivered online via Zoom. Following referral and matching of the peer supporter and the person being supported, collaborative decisions were made between the person being supported and the peer supporter regarding the focus, the location and duration of support.

The aim of this study is to examine and evaluate the effectiveness and impact of the “For Survivors, By Survivors” pilot cancer survivorship program for the peer supporter, the person being supported and the line manager of the peer supporter.

## 2. Methods

### 2.1. Design

The program was evaluated after its completion without the use of pre-test measures or a control/comparison group. Therefore, a one-group post-test-only design was deemed most appropriate. The Practical, Robust, Implementation and Sustainability Model (PRISM), incorporating the Reach, Effectiveness, Adoption, Implementation and Maintenance (RE-AIM) Outcomes Framework guided the research team in defining the study variables [8]. PRISM and RE-AIM are widely used to evaluate public health and implementation initiatives [9]. PRISM was used to ascertain the multilevel contextual factors that influenced the pilot program such as organizational readiness, systems and resources available or needed (training, funding, monitoring). RE-AIM helped assess how well the program worked. The Strengthening the Reporting of Observational Studies in Epidemiology (STROBE) checklist was used to guide the reporting of this study [10] (S1 Checklist).

### 2.2. Sample and setting

Participants were eligible to participate if they were peer supporters, peers (i.e., individuals being supported), and line managers of peer supporters. Participants were recruited via email by the Marie Keating Foundation – an Irish cancer charity focused on cancer prevention, early detection, education, and support services for those affected by cancer – that acted as a gatekeeper. This was to ensure recruitment was appropriate and that participants’ identities were protected. All participants were employees of the Irish health service and working within the pilot area which encompassed 19 of 32 counties.

According to guidelines for pilot studies, sample sizes between 24 and 50 participants are considered adequate to identify potential issues with study design, recruitment, and intervention delivery, and to provide variance estimates for future larger-scale studies [11,12]. Moreover, similar pilot evaluations in supportive cancer care have used comparable sample sizes [13,14]. Therefore, we aimed to recruit up to 50 participants. A total of 42 participants were ultimately included in the study, which was considered sufficient to meet the study objectives.

### 2.3. Procedures

Participants were issued a specified link to direct them to the appropriate survey. Links were embedded in an email from a gatekeeper within the Marie Keating Foundation. This included a participant information leaflet and consent form embedded in the Qualtrics survey platform, which participants completed prior to accessing the surveys. Participants were invited to complete the surveys if they had (i) undertaken Peer Supporter training, (ii) had a Peer Support interaction (as peer supporter or peer) or (iii) were peer supporters’ line managers. No identifying information appeared on the survey. Demographic information was collected but not assigned to survey responses. Participants were advised in the participant information leaflet that they could withdraw participation at any time up to two weeks after completion of survey data. Data were anonymized using a user generated code.

Data were collected from 23^rd^ October 2024–22nd January 2025 via the Qualtrics survey platform. Survey data were stored electronically on Qualtrics with access granted to the research team with institutional login details only. The responses were anonymized to reduce response bias and encourage more genuine responses without fear of work-related consequence [15].

### 2.4. Data sources and measurement

Data were collected using three distinct researcher-designed surveys (Table 1). These were adapted from Tolins et al [16] who evaluated feedback from a Peer Outreach Support Team in terms of the training received and overall evaluation of awareness and adoption of the training. These surveys were adapted to better reflect the overall aims of our study and reflect each of the three cohorts’ experience and role within the “For Survivors, By Survivors” peer support program. As part of instrument development, face validity was established by piloting the surveys within the research team, focusing on clarity, ease of completion, and alignment with the study objectives. The letter “P” is used to present survey results from people being supported, (peers), “PS” for peer supporters, and “LM” for line managers.

**Table 1:** Survey questions for each participant group.

Population	Source	Number of Items	Answer Options	Scoring
Peer Supporters	Researcher designed, adapted from Tolins et al [9]	10 items divided as follows:		
		(i) Overall rating of the program	Item assessed on a 5-point Likert scale	Answers range between “Excellent=1; Good=2; Neutral=3; Poor=4; Very Poor=5”
		(ii) Received a framework to use in practice	Item assessed on a 3-point ordinal scale	Answers range between “Yes=1; Maybe=2; No= 3”
		(iii) Navigate the available resources for assisting colleagues	Item assessed on a 5-point Likert scale	Answers range between “Strongly Agree=1; Agree=2; Disagree=3; Disagree =4; Strongly Disagree =5”
		(iv) Understanding the role as a peer supporter	Item assessed on a 5-point Likert scale	Answers range between “Extremely well=1; Somewhat well=2; Neutral=3; Poorly=4’ Extremely poorly=5”
		(v) Receiving peer support interaction in the past	Item assessed on a 3-point ordinal scale	Answers range between “Yes=1; No=2; No, but reached out=3”
		(vi) Overall satisfaction of the training program	Item assessed on a 5-point Likert scale	Answers range between “Very unsatisfied=1; Unsatisfied=2; Neutral=3; Satisfied=4; Very satisfied=5”
		(vii) Overall satisfaction of the preparation for their role as mentor	Item assessed on a 5-point Likert scale	Answers range between “Very unsatisfied=1; Unsatisfied=2; Neutral=3; Satisfied=4; Very satisfied=5”
		(viii) Recommendation of the training program to other potential peer support mentors	Item assessed on a 4-point Likert scale	Answers range between “Yes, would highly recommend = 1; Yes, would recommend = 2; Neutral = 3; No, would not recommend = 4
		(ix) Potential barriers to the participation as a peer support mentor	Item assessed on a multiple options answer format	N/A
		(x) Open ended questions (4 items)	N/A	N/A
Line managers	Researcher-designed, adapted from Tolins et al [9]	7 items divided as follows:		
		(i) Awareness of cancer survivorship peer support service	Item assessed on a 3-point ordinal scale	Answers range between “Yes=1; Maybe=2; No=3”
		(ii) Referral to the program	Item assessed on a binary choice	Answers range between “Yes=1; No=2”
		(iii) The impact of cancer survivorship peer support service	Item assessed on a 5-point Likert scale	Answers range between “Very positive impact=1; Somewhat positive impact=2; Neutral =3; Somewhat negative impact=4; Very negative impact=5”
		(iv) Overall satisfaction of the program	Item assessed on a 5-point Likert scale	Answers range between “Very unsatisfied=1; Unsatisfied=2; Neutral=3; Satisfied=4; Very satisfied=5”
		(v) Would recommend the program to other departments	Item assessed on a 5-point Likert scale	Answers range between “Yes, would highly recommend =1; Yes, would recommend= 2; Neutral=3; No, would not recommend=4”
		(vi) Barriers to limit your referral of others/ Acceptance of program as a resource for employees	Item assessed on a multiple options answer format	N/A
		(vii) Open ended question (1 item)	N/A	N/A
Peers	Researcher-designed, adapted from Tolins et al [9]	12 items divided as follows		
		(i) Having peer support interaction in the past	Item assessed on a binary choice	Answers range between “Yes=1; No=2”
		(ii) The helpfulness of peer support interactions	Item assessed on a 5-point Likert scale	Answers range between “Very helpful=1; Helpful=2; Somewhat helpful=3; Not at all helpful=4; Negatively impacted me=5”
		(iii) Changes in emotional well-being	Item assessed on a 5-point Likert scale	Answers range between “Significant improvement=1; Some improvement=2; No change=3; Some decline=4; Significant decline=5”
		(iv) Additional support resources provided by peer supporter	Item assessed on a 5-point Likert scale	Answers range between “Yes = 1; No = 2
		(v) Changes in comfort with talking about feelings/emotions	Item assessed on a binary choice	Answers range between “Much more comfortable=1; Somewhat comfortable=2; No change in comfort=3; Less comfortable=4”
		(vi) Satisfaction of interactions with peer supporter	Item assessed on a 4-point Likert scale	Answers range between “Very unsatisfied=1; Unsatisfied=2; Neutral=3; Satisfied=4; Very satisfied=5”
		(vii) The effectiveness of the peer support service	Item assessed on a 5-point Likert scale	Answers range between “Very effective=1; Effective=2; A little effective=3; Neutral=4; Not effective=5”
		(viii) Overall satisfaction of the program	Item assessed on a 5-point Likert scale	Answers range between “Very unsatisfied=1; Unsatisfied=2; Neutral=3; Satisfied=4; Very satisfied=5”
		(ix) The recommendation of the program to other employees potentially needing support	Item assessed on a 4-point Likert scale	Answers range between “Yes, would highly recommend =1; Yes, would recommend= 2; Neutral=3; No, would not recommend=4”
		(x) Barriers to limit the participation of the program	Item assessed on a multiple options answer format	N/A
		(xi) Understanding of the resources, the ability to seek help, and self confidence in being at work	Items assessed on a 7-point Likert scale	Answers range between “1 to 7” 1 = the lowest 7 = the highest
		(xii) Open ended questions (5 items)	N/A	N/A

To assess reach, all who engaged with the evaluation were asked to complete an anonymized record of whether support interaction occurred, the number of interactions, the length of time of the interactions, and whether there were offers of interaction that were declined. Peer supporters rated the training received and confidence to be a peer supporter having completed the training. Peers rated the overall satisfaction with the program, including access to further support/resources and any changes in psychological well-being and ability to discuss their diagnosis and needs. Line Managers considered their awareness of and use of the peer support program. Overall satisfaction and impact of the program was explored.

The survey included both categorical and open-ended questions designed to probe participants overall impression of the program, their confidence in navigating resources, perceived knowledge acquisition, and their understanding of the program referral process.

### 2.5. Statistical analysis

Data were cleaned using SPSS Version 29.0 [17] and statistical analyses were performed in R (version 4.4.2 [18]). Descriptive statistics for categorical variables were presented as numbers and proportions. To illustrate uncertainty in the estimates a 95% confidence interval (CI) was provided for proportions using the Wilson approach [13]. This approach has been recommended for small sample sizes [14] and was available in the R package “DescTools” [19]. The statistical significance of differences between peer supporters, line managers and peers were assessed using Fisher's exact test (“fisher.test” in R [18]).

Continuous/ordinal variables including meeting duration and 7-point Likert scales were described using the median and interquartile range as well as minimum and maximum values. The 7-point Likert scales represented peers’ abilities, confidence and understanding before and after the intervention. The statistical significance of improvements in these Likert scores (before versus after the intervention) were assessed using an approximative Wilcoxon-Pratt Signed-Rank Test available in the R package “coin” [20]. This method used Monte Carlo resampling (100,000 resamples) which can be superior to other approaches for sample sizes ≤ 20 [21]. These comparisons were presented using boxplots. Effect sizes were calculated using the formula r = Z/ √N) with values close to 0.1 being interpreted as a “small” effect, 0.3 as “medium” and 0.5 as “large”.

### 2.6. Ethics

Ethical approval was granted by the Social Research Ethics Committee at University College Cork (2024-084A1) and the Health Service Executive's Research Ethics Committee (RRECB0624). Consent was obtained electronically prior to participants proceeding to survey. The information leaflet and the end of the survey contained contact details of researchers and verified support services. This was deemed necessary since talking about personal experiences with cancer can be distressing [3].

## 3. Findings

### 3.1. Sample characteristics

A total of 50 attempts were made at the survey. Eight incomplete surveys were excluded from the analysis due to 60% or more missing data. This led to a sample size of 42 participants including 19 peer supporters, 15 peers, and 8 line managers. Participants were predominantly female (n = 36, 84%), and all were 30 years of age or older. The characteristics of survey participants are presented in Table 2.

**Table 2:** Characteristics of survey participants (n = 42).

		Peer supporters (N = 19)		Peers (N = 15)		Line managers (N = 8)		Total (N = 42)	
Variable	Values	Number	Percentage	Number	Percentage	Number	Percentage	Number	Percentage
**Age (years)**	**18–29**	0	0%	0	0%	0	0%	0	0%
	**30–39**	0	0%	0	0%	1	13%	1	2%
	**40–49**	6	32%	4	27%	0	0%	10	24%
	**50–59**	11	58%	9	60%	5	63%	25	60%
	**≥ 60**	2	11%	2	13%	2	25%	6	14%
**Gender**	**Female**	18	95%	12	80%	6	75%	36	86%
	**Male**	0	0%	3	20%	2	25%	5	12%
	**Prefer not to say**	1	5%	0	0%	0	0%	1	2%
**Profession/ Role**	**Administration**	3	16%	2	13%	1	13%	6	14%
	**Environmental Protection**	2	11%	0	0%	2	25%	4	10%
	**Health Promotion**	1	5%	2	13%	0	0%	3	7%
	**Management**	2	5%	1	7%	1	13%	4	10%
	**Medicine**	0	0%	0	0%	1	13%	1	2%
	**Health or Social Care Professionals**	1	5%	1	7%	0	0%	2	5%
	**Nursing**	7	37%	7	47%	2	25%	16	38%
	**Teaching**	1	5%	0	0%	0	0%	1	2%
	**Support**	0	0%	1	7%	1	13%	2	5%
	**Prefer not to say**	2	11%	1	7%	0	0%	3	7%

### 3.2. Analysis

Analyses of the surveys of all three cohorts were conducted separately. The descriptive statistics provided a clear overview of participants’ responses, revealing notable trends in the data. Similar findings were then categorized under the following four broad categories: i) Peer to Peer Interactions ii) Barriers and Challenges to Program Participation iii) Program Satisfaction and Effectiveness and iv) Future Recommendations and Improvements.

Data from three open-ended questions pertaining to participants’ perceptions of the program, and recommendations for the program were analyzed using inductive content analysis [22]. Data were read multiple times for immersion, then open coding was used to identify meaningful units of text which were subsequently categorized. Two researchers reviewed codes independently to ensure reliability.

### 3.3. Peer to peer interactions

For the sample of 19 peer supporters, 16 (84%) had a peer support interaction, two (11%) had reached out to a colleague but they had declined, and for one (5%) an opportunity did not arise. For the sample of peers all 15 (100%) reported an interaction. As illustrated in Table 3, most peer supporters (n = 11, 69%) and most peers (n = 8, 53%) had only one interaction. The most interactions lasted 5–15 minutes (n = 10, 40%) with a range of 5–180 minutes according to peer supporters and most were 20–30 minutes (15, 50%) range 5–60 minutes. The total time spent on interactions ranged between 10 and 180 minutes for peer supporters with a median of 52.5 minutes and an interquartile range (IQR) of 30–60 minutes. For peers it ranged between 10 and 120 minutes with a median of 60 minutes (IQR: 18.75–80). Of note, the discrepancy between peers and peer supporters arises from the research design, and not matched or paired sampling.

**Table 3:** The number and durations of interactions reported by the samples of peer supporters and peers.

Duration each (total duration)	Number of peer supporters (%)	Number of peers (%)
**Had 1 interaction**	**11 (69%)**	**8 (53%)**
10 minutes (10)	1 (6%)	1 (7%)
15 minutes (15)	–	1 (7%)
15–20 minutes (17.5)	–	1 (7%)
20 minutes (20)	1 (6%)	1 (7%)
30 minutes (30)	1 (6%)	–
40 minutes (40)	–	1 (7%)
45 minutes (45)	2 (12%)	–
40–60 minutes (50)	1 (6%)	–
50–60/ 55 minutes (55)	3 (19%)	–
60 minutes (60)	1 (6%)	3 (20%)
180 minutes (180)	1 (6%)	–
**Had 2 interactions**	**4 (25%)**	**2 (13%)**
5–7 minutes (12)	–	1 (7%)
10 minutes each (20)	1 (6%)	–
15 minutes each (30)	1 (6%)	–
30 minutes each (60)	1 (6%)	1 (7%)
5 and ≥120 minutes (125)	1 (6%)	–
**Had 3 interactions**	**–**	**2 (13%)**
15 minutes (45)	–	1 (7%)
30–40 minutes (105)	–	1 (7%)
**Had 4 interactions**	–	**3 (20%)**
20–30 minutes (100)	–	1 (7%)
30 minutes (120)	**–**	2 (13%)
**Had 6 interactions**	**1 (6%)**	–
Two 30 minutes & four 15 minutes (120)	1 (6%)	–

### 3.3. Barriers and challenges to program participation

The most common barrier limiting participation in the program was cited as “lack of time” across all groups, reported by 60% (n = 9) of peers, 58% (n = 11) of peer supporters, and 50% (n = 4) of line managers (indicating no difference between the groups, Fisher's exact *p*-value = 0.923). Overall, for all groups the percentage was 57% (95% CI: 42%–71%). Peers reported concerns with *“departmental workload”* and *“discomfort discussing feelings with colleagues.”* Peer supporters and line managers were concerned that more training was needed for peer supporters. Peer supporters reported challenges related to a lack of confidence when providing information to their colleagues due to medico-legal concerns. Peers, in the free text options, noted concerns relating to the need for individualized support. *(“What works for one person might not work for another but with different outlets, a person will hopefully find one that suits them.”)* and the lack of certainty relating to work attendance or ability to participate in the program *(“During treatment there can be many off or sick days”).*

### 3.4. Program satisfaction and effectiveness

Most participants found the program satisfactory. For peer supporters, 84% (n = 16) were “satisfied” or “very satisfied” with the training program and their role preparation. All peer supporters would recommend the intervention (100%, n = 19) to other potential peer supporters. Among line managers, 88% (n = 7) indicated that they were “highly satisfied” or “very satisfied” with the program in their department and considered recommending it (100%, n = 8) to other departments. For peers 80% (n = 12) expressed satisfaction, and 93% (n = 14) would recommend the program to other employees who may potentially need support. Thus, amongst all participants, 83% (95% CI: 69%−92%) were satisfied with the program and 98% would recommend the program (95% CI: 88%−100%).

Peers (n = 15) were asked to indicate any changes in their emotional well-being following peer support interactions on a 5-point Likert scale (1-significant decline to 5-significant improvement), with 27% (n = 4) reporting no change and 73% (n = 11) indicating improvement.

The helpfulness of peer support interactions was rated on a five-point Likert scale (1-negatively impacted to 5-very helpful). There was a notably positive response, with 53% of peers indicating that their interactions were very helpful (n = 8), 27% describe interactions as helpful (n = 4) and 13% (n = 2) as somewhat helpful, while 7% (n = 1) found them not at all helpful. No participant felt that the interactions had negative effects.

After peer support interactions 73% (n = 11) of peers felt more comfortable discussing work-related emotions in general, with 27% (n = 4) reporting no change in their self-reported comfort. No peers reported negative changes to their comfort in discussing work related emotions. Interestingly, 13% (n = 2) expressed dissatisfaction with their interaction(s) with their peer support mentor, while 7% (n = 1) felt neutral, and 80% (n = 12) reported satisfaction. Free text was provided for sharing feelings regarding the interactions. Four participants (27%) had no further comments, while other peers (n = 7, 47%) gave predominantly positive feedback. Two participants (13%) found the support particularly useful for practical advice and suggestions for further support, although one felt it offered limited resources for post-treatment work support. Peer supporters were described positively, being recognized as dedicated, empathetic, and easy to converse with, *“I found my peer support person easy to talk to, empathetic, and providing practical advice.”* (P). This resulted in the program being a very positive experience for most, *“I feel very lucky to have accessed this service. It was one of the most helpful aspects of my treatment”* (P). Participants also enjoyed hearing the peer supporters’ stories and discussing shared interests.

All peer supporters (n = 19) rated the peer support training program positively, with 32% (n = 6) responding “Good” and 68% (n = 13) responding “Excellent.” All responses indicated that participants received a framework for providing peer support to colleagues. Every participant either “agreed” or “strongly agreed” that they were able to navigate the resources available for colleagues returning to work after a cancer diagnosis.

Peer supporters selected either “somewhat well” or “extremely well” when asked if they understood their role as peer supporters after training. The most influential components of the training included communication with other peer supporters (n = 14, 74%), role-play scenarios (n = 8, 42%), and speaker presentations (n = 5, 26%). Two participants (n = 2,11%) stated that they found all aspects of the training helpful.

*“Personally, it was extremely beneficial for me to realize that my feelings were very normal and similar to those of others’ lived experiences. This understanding has helped me and will continue to assist me when supporting others who may feel similarly.” (PS)*

### 3.5. Future recommendations and improvements

Participants were asked if there were changes that they would recommend to the program overall, citing a lack of access and awareness of the program as well as more of an individualized approach. For example, three peers indicated that more advertisement is needed for the program *(“Perhaps more awareness of the program, leaflets/videos etc that explain the program so that people have a little more clarity about the program provision.” “Advertised more widely. Very few individuals I met heard about the program.”* One line manager and three peer supporters suggested that more awareness is needed about the program *(“While a member of our staff participates as a peer survivor, I am not sure what their role involves or what happens when someone is referred. I am sure I could find out this information by speaking to the member of staff, and i have just now googled it and found out – however, I would say most staff including line managers, don’t necessarily know much about it.” (LM)) (“I’d just like it to be more well known about the service so that people could/ would access it- I feel like the more people could be referred the better it would make supporters skills.” (PS)).*

Peer supporters noted that the face-to-face elements of the training were more beneficial than the online components, with 42% (n = 8) using free text to express a preference for in-person training. For example, the opportunity to connect with other peer supporters and practice skills in person positively influenced the perception of the training delivered. Additionally, 26% (n = 5) of participants mentioned that the time gap between training and implementation could be improved, stating, *“I think because I was part of the first group trained, it seemed slow to get moving. I first applied in 2021, and it is now 2024, and we are only really up and running now.”* A summary of survey responses within these categories is presented in Table 4.

**Table 4:** Summary of survey responses for three cohorts (peers, peer supporters, line managers).

Item	Answer options	Line managers n (%)	Peer supporters n (%)	Peers n (%)
Peer to Peer Interactions	Had an interaction	–	16 (84%)	15 (100%)
	Did not have an interaction	–	3 (16%)	0 (0%)
	Made a referral	3 (38%)	–	–
	Did not make a referral	5 (62%)	–	–
Barriers to limit the participation of the program *	Lack of time	4 (50%)	11 (58%)	9 (60%)
	Our department is too busy	0 (0%)	9 (47%)	2 (13%)
	People are not comfortable talking about their feelings with colleagues	1 (12%)	0%	2 (13%)
	More training of peer supporters is needed	1 (12%)	5 (26%)	2 (13%)
	Medico-legal concerns	0 (0%)	2 (11%)	0 (0%)
	Other	3 (38%)	6 (32%)	5 (33%)
Overall satisfaction and effectiveness of the training program	Overall satisfied with the peer support program	7 (88%)	16 (84%)	12 (80%)
	Neutral with the peer support program	1 (12%)	1 (5%)	1 (7%)
	Unsatisfied with the peer support program	0 (0%)	2 (11%)	2 (13%)
	Peer support interactions were helpful	–	–	14 (93%)
	Peer support interactions were unhelpful	–	–	1 (7%)
	Improvement in emotional wellbeing	–	–	11 (73%)
	No change in emotional wellbeing	–	–	4 (27%)
	Decline in emotional wellbeing			0 (0%)
	More comfortable talking about work-related feelings	–	–	11 (73%)
	Did not change comfort talking about work-related feelings	–	–	4 (27%)
	Less comfortable talking about work-related feelings	–	–	0 (0%)
	Satisfied interactions with peer supporter	–	–	12 (80%)
	Neutral interactions with peer supporter	–	–	1 (7%)
	Unsatisfied interactions with peer supporter	–	–	2 (13%)
	Peer supporter program was good	–	19 (100%)	–
	Peer supporter program was poor	–	0 (0%)	–
	Received a framework to use in practice	–	19 (100%)	–
	Not received a framework to use in practice	–	0 (0%)	–
	Agree that they can navigate the available resources for assisting the colleagues	–	19 (100%)	–
	Not agree that they can navigate the available resources for assisting the colleagues	–	0 (0%)	–
	Understanding the role as a peer supporter was well	–	19 (100%)	–
	Understanding the role as a peer supporter was poor	–	0 (0%)	–
The most influential part of the training	Communication with other peer supporters	–	14 (74%)	–
	Role play	–	8 (42%)	–
	Presentations form speakers	–	5 (26%)	–
	Education around self-care	–	2 (11%)	–
	Found it all helpful	–	2 (11%)	–
	Resource materials	–	1 (5%)	–
Recommendation of the program	Yes	8 (100%)	19 (100%)	14 (93%)
	No	0 (0%)	0 (0%)	1 (7%)

*Participants could select more than one response.

### 3.6. 7-point Likert scales for peers

**Fig 1:**
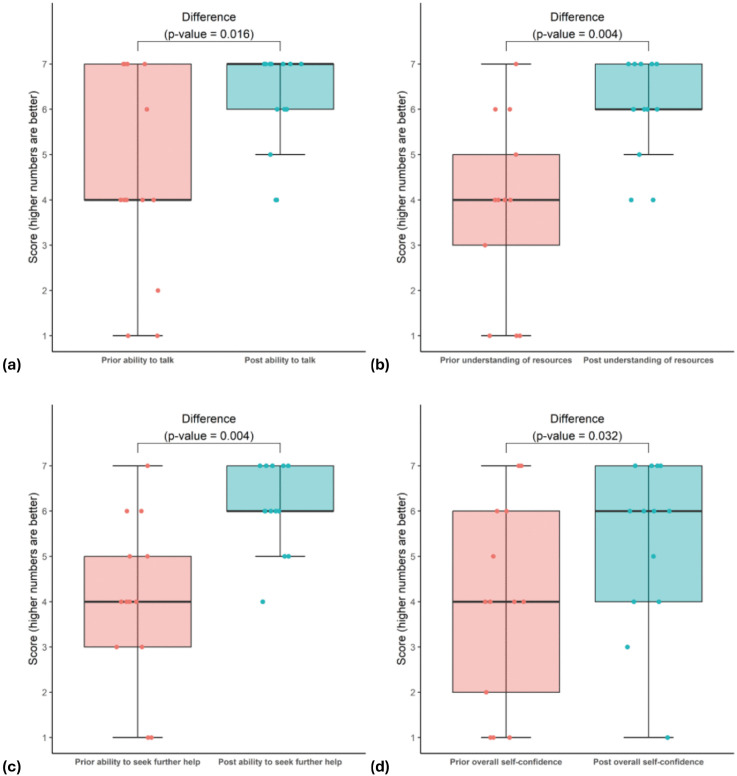
Boxplots illustrating the peers’ scores on 7-point Likert scales representing before and after the intervention including: (a) their ability to talk, (b) their understanding of resources available, (c) their ability to seek further help and (d) their overall self-confidence.

For peers, 7-point Likert scales were used to assess their self-reported levels of ability, confidence and understanding before and after interactions with a peer supporter (Fig 1). Changes in pre- and post-intervention Likert scale scores were analysed using an approximate Wilcoxon-Pratt Signed-Rank Test due to the ordinal nature of the data and non-normal distribution assumptions, as well as the small sample size. As illustrated in Fig 1 they reported statistically significant improvements (*p*-values < 0.05) in all four categories with the median scores increasing from 4 representing before the intervention to either 6 or 7 for after the intervention. These improvements included their self-rated ability to talk (median score increased from 4 to 7, *p*-value = 0.016), their self-rated understanding of the resources available (median score 4–6, *p* = 0.004), their self-rated ability to seek further help (median score 4–6, *p* = 0.004) and their overall self-confidence (median score 4–6, *p* = 0.032). The effect sizes (r) for these four improvements were all large being 0.72, 0.80, 0.80, and 0.67, respectively.

### 3.7. Open-ended question categories

All 42 participants (line managers, n = 8; peer supporters, n = 19; peers, n = 15) were asked, *“Is there anything you would like to tell us about your perception of or experience of the cancer survivorship peer support service “For Survivors, By Survivors” overall?”.* Peer supporters and peers were asked, *“Is there anything you would like to tell us about changes you would like to see made to the program overall?”* Peers were asked, *“Can you please share why you found your interaction with a peer supporter helpful or unhelpful?”*

Participants stated the program was *“necessary”* (LM), *“much needed”* (LM) and overall described their experiences with the program as *“positive”* (P) and *“appreciated”* (P). Peer supporters found the role *“rewarding”,* with *“personal benefits”* and commented on the usefulness of the training to prepare them for the role of befriending and signposting colleagues. Two main categories were identified from the data: Program ethos: supporting and connecting and Program recommendations and changes.

### 3.8. Category 1: Program ethos: Supporting and connecting

The program ethos was described as “*flexible*” (P), “*relatable*” (P) and “*interesting*” (P). Because of its peer status, the value of a *“shared experience”* (PS), where someone *“knows what it’s like”* (P) was noted by participants. The ability of peer supporters to *“guide”* (P), *“signpost”* (PS), *“navigate resources”* (PS) and meet the person *“where I am; doesn’t push towards the future”* (P), reflected the ethos of the program.

Giving or receiving support was considered central with all participants commenting on this aspect of the program. Line managers (21%) noted *“support for employees”* as being important while three peers valued the *“advice and information”* (P) (20%), the emotional support provided, where they felt “*listened to without being told what to do” or “being judged” (8*%). The perception from peer supporters was that the program was *“supportive and informative”* (5%), particularly the *“practical support”* and it helped in *“understanding needs”* (5%).

Making connections was another meaningful aspect of the program. One line manager noted the *“peer connection”* as being significant, with this reflected by peers who favoured “*shared interests”* (8%) and *“connection”* (15%). The “*empathy and understanding of the peer supporter*” (P) was the most positive aspect of the interaction for those being supported (33%). The dimensions of “*relating through lived experience*”, (P) and “*feeling heard and less alone*” (P) were reported by peers as equally helpful (20%). Peer supporters also commented on this connectivity, identifying their role as *“rewarding”* (PS) and how they personally *“benefited from being involved”* (PS) because of their interactions with other peer supporters and their peers.

Some limitations and unmet needs were noted. Thirteen per cent of peers indicated that there is a “*need for more than listening*”, and “a *lack of relevant support or information*”. Another person being supported stated there was no new information made available. One line manager noted a *“lack of clarity”* about the peer supporter role and program awareness. This was reflected in peer supporters’ reflections on *“role uncertainty”* (PS) (5%), *“training length” (*PS) (5%) and *“follow-up ambiguity”* (PS) (5%) as potential difficulties in relation to the program.

### 3.9. Category 2: Program recommendations and changes

Participants reflected on future iterations of the program. More advertising of the program was recommended by peer supporters and peers (21%; 8%). Both peers and peer supporters also noted a need for *“wider availability”* of the program beyond the HSE (8%; 11%). Peers also suggested the need for greater “*financial guidance”* (P) (8%) on how to obtain financial aid during treatment. *“More tailored support”* (8%) that prioritizes individuals’ specific difficulties was recommended by peers for inclusion in the peer support program. Peer supporters commented on the importance of recording informal interactions to reflect their peer support role beyond the formal program (i.e., providing peer support to people who have not been formally referred to the program) (5%).

Proposed changes to “*how the training is* delivered” (PS) were made by peer supporters (32%). It was recommended that training be conducted face-to-face, consist of shorter days, and occur more frequently with *“more meet ups or informal chats with other peer supporters”* (PS). Table 5 summarizes the overall findings of open-ended questions asked of three groups about the cancer peer support program

**Table 5:** Open-ended question categories and subcategories.

Main categories	Subcategories	Codes
Program Ethos: Supporting and Connecting	Perception of the program	Felt lucky to access it, one of the most helpful aspects of treatment (P) Program is much needed due to staff numbers and nature of HSE (LM) Helping colleagues is rewarding (PS)
	Program approach as supportive	Flexible gentle check-ins every 2–3 weeks (P) Very good support for employees (LM) Helping with navigation of resources/services (PS)
	Peer connection	Very empathetic and easy to talk to (P) People like to speak to survivors who have similar experiences (LM) Connection with others with similar lived experience (PS)
	Program limitations	Need for more than listening (P) Uncertainty about the peer supporter role and awareness (LM) Role uncertainty/Not sure if I should follow-up if no contact (PS)
Program Recommendations and Changes	Advertising and expanding the program	Needs more awareness/promotion campaigns (PS) Record support given to those outside the formal program (PS) Advertise more widely, few individuals know about the program (P)
	Meeting needs	Peer support could be more tailor-made if specific difficulties shared (P) More information on how to get financial aid during treatment (P)
	Delivery of training	Training was to listen, wanted more support (P) More frequent training (LM) Training needs to be face to face/shorter sessions (PS)

## 4. Discussion

The study aimed to examine and evaluate the effectiveness and impact of a pilot cancer survivorship peer support program, *“*For Survivors, By Survivors.*”* The evaluation was guided by the PRISM framework, examining contextual factors, and RE-AIM components that influenced how the program operated in a real-world healthcare setting [23]. Perspectives on the program were gathered to understand its relevance, strengths and potential for expansion. Being a pilot, the program faced restrictions imposed by organizational systems and the work environment (e.g., limited access to global email communications to advertise the program’s availability, lack of participant time). These restrictions affected its reach, adoption and implementation. All participant cohorts (peers, peer supporters, line managers) identified the limited program awareness, and subsequent access, as a significant challenge that would need to be addressed if the program was to be expanded. These limitations may have impacted access to the program for individuals returning to work post cancer treatment.

Prior to commencing their role as peer supporters, participants undertook specific training. This training did meet most of their needs, however, the time lapse between training and commencing the role was an issue. The restricted in-person training and continuous professional development opportunities were also noted, potentially influencing how peer supporters perceived the degree of organizational support for the initiative. These factors shed light on the organizations’ readiness to implement the program, and system and resource limitations, that influence a program’s success [8,23]. Nonetheless, the health service initiated the program development, demonstrating a commitment to the health and well-being of their employees [24]. It can be argued that a recognition of the emotional strain, workload pressure and potential stigma or isolation associated with cancer survivorship and return to work was evident [25]. The introduction of the peer support program sits with the core concepts described by the Jobs Demands-Resource (JD-R) Model [26]. The model integrates two basic psychological processes, a stress process and a motivational process. Every job has its own demands and resources and the balance between these influence employee outcomes like burnout, engagement and productivity [27]. Excessive job demands and lacking resources (stress process) may lead to negative outcomes such as sickness or poor motivation. A motivational process, triggered by abundant job resources can result in positive outcomes including employee safety, intention to stay and organizational commitment. By increasing resources such as social support such as peer support, and reducing high job demands, burnout can be prevented, adaptation and work engagement enhanced [28–30].

Returning to work, after a cancer diagnosis in particular, poses challenges for employees [31]. Unmet supportive care needs are well-documented in the literature on cancer survivorship, including psychological, informational and physical needs [5,32,33]. Addressing any potential unmet needs can positively impact coping ability for employees, enhancing engagement and outcomes [29,34]. Peer support programs have been shown to help address supportive care needs by offering timely tailored interventions that bring relief, increase knowledge, understanding, and a sense of connection and belonging [35–37]. Support that includes information on practical issues (e.g., financial guidance during sick leave) is valued [38] and emotional reintegration as part of a person’s return to work can be impactful [39,40].

In this study, peers perceived emotional support, practical help, and increased psychological safety (i.e., more comfortable to discuss their feelings in the workplace) as outcomes of their peer support interaction. These findings align with the JD-R model [27] where an initiative was introduced in recognition of employee needs and to mitigate work demands with additional resources. This highlights the potential value of structured peer support to foster a healthier work environment. Likewise, peer supporters described personal growth and a sense of reward from participating in the program. Line managers commented on the necessity of the program in supporting colleagues who had been diagnosed with cancer. They also expressed their willingness to recommend the program to other departments. These contextual or hidden impacts are evidenced elsewhere in the literature where the “ripple effect” of support enhanced well-being and relationships within the workplace while increasing confidence beyond this setting [41]. This study adds to the limited research in this area.

Participants in our study were asked to consider the maintenance and sustainability of the program. Participants emphasized the need for increased publicity, better communication, and clearer referral processes. The importance of tailored interventions was identified as key, as was the approach of the peer supporter, and the delivery of training going forward. As stated, peer supporters responded positively to the training and overall program, although many identified a gap between the content of the training and the realities of actual peer support interactions, suggesting a need for ongoing support or continuing practice development. This has a cost implication that would need to be addressed to allow the program to evolve. Promoting awareness and addressing financial or logistical challenges are reported elsewhere if programs are to succeed [42–44]. Other research has emphasized how organizational support is crucial for reintegration [40,45]. The necessity of balancing implementation fidelity and adaptation for effective program delivery has been identified in the literature [23,46].

The study has implications for practice, policy and future research. The program reflects a policy level recognition of the emotional and practical challenges faced by cancer survivors returning to work. It aligns with broader frameworks such as the International Labour Organization [47] inclusion of health and safety as fundamental rights for workers. Policies should ensure clearly defined peer supporter roles, concise, regular training sessions, protected time during work hours for participation, and earlier program initiation, all reported in the literature to increase maintenance [48–50]. For broader implementation, policies must support integration into workplace culture and systems (e.g., induction processes, back-to-work interviews). Practice implications focus on training and support, awareness and communication, tailored interventions and managerial involvement. While initial training was well received, gaps between training and real-world application suggest a need for ongoing professional development, hybrid or refresher training models and access to peer learning and expert support. Peer support should be personalized, with attention to emotional, practical, and psychological needs. Line managers should play a key role in endorsing and facilitating the program, which can enhance uptake and impact. Future studies should assess long-term outcomes of peer support programs, including their impact on return-to-work success, well-being, and organizational culture. The study supports the use of the Job Demands–Resources model to understand how peer support can buffer stress and enhance engagement. Further research in this area is warranted into the broader, less visible impacts of peer support, such as improved team dynamics and psychological safety.

## 5. Limitations

This study’s one-group post-test-only design limits causal conclusions due to the absence of baseline data and a control group. Consequently, it is difficult to determine whether the observed outcomes can be attributed to the program or to other unknown contextual factors. The small sample size reduces statistical power and generalizability. Reliance on self-reported data may introduce social desirability bias and recall bias. To assess the program’s maintenance a longitudinal component is recommended, across different settings with data collections at specified intervals (e.g., six monthly). Given the program’s pilot status, broad organizational communication strategies could not be used to extend reach and adoption.

While only face validity was established, instrument design was informed by existing surveys and tailored to the study’s specific aims. Future research could build on this foundation by formally validating the instrument in a broader sample.

Only participants who were interested in participating completed the survey, increasing the risk of self-selection bias. Finally, the gender distribution of respondents was skewed toward those who identified as women, potentially limiting the transferability and generalizability of results to male identifying and gender-diverse individuals. Future research should adopt [51,52] a gender expansive lens to enhance understanding of needs and potential barriers to help-seeking.

## 6. Conclusion

The purpose of the study was to evaluate a pilot cancer survivorship peer support program within the workplace, “For Survivors, By Survivors”. Findings indicate strong support for its broader implementation. The peer support program positively impacted those receiving support, those providing it, and the organization (evaluated through line manager responses). Despite these benefits, the program faced barriers to wider engagement. A lack of program awareness and time constraints emerged as significant issues for participants. Peer supporters also identified the training to practice gap as undermining their confidence. These findings underscore environmental and structural influences that may require organizational intervention to address.

While peer support is an effective and well-received approach, its success depends on better integration into the workplace culture, improved communication about its availability and purpose, and a reduction of systemic barriers. Future program development should focus on strategies to streamline the peer supporter training and progression, increased visibility, accessibility, and governance. Future research should prioritize longitudinal evaluations of peer support programs implemented at a larger scale to better assess their effectiveness.

## Supporting information

S1 Checklist The Strengthening the Reporting of Observational Studies in Epidemiology (STROBE) Checklist. (DOCX)

S2 Checklist The TiDieR (Template for Intervention Description and Replication) Checklist. (DOCX)
