# Supplementary material for: Evaluation of ‘For survivors by survivors’, a cancer survivorship peer support pilot program for healthcare staff: A one-group post-test-only study
Source: PLoS One. 2025 Dec 2;20(12):e0337591. doi: 10.1371/journal.pone.0337591 (PMC12671735; doi:10.1371/journal.pone.0337591)
Supplement: S2 Checklist — (DOCX) [file pone.0337591.s002.docx]

**S2 Checklist: The TiDieR (Template for Intervention Description and Replication) Checklist**

| **BRIEF NAME OF INTERVENTION** | Peer supporter training programme |
| --- | --- |
| **WHY** | **Rationale:** The rationale behind the intervention is based on evidence showing that peer support plays a vital role in cancer survivorship. Peer support facilitates emotional validation, understanding, practical advice and a sense of belonging, helping survivors to cope with the challenges of recovery and re-engaging with the workplace. Peer supporters need training and ongoing support to deliver the service.  **Aim:** The training aimed to provide a psychologically safe space for trainees i) to understand survivorship, ii) explore their personal cancer journey, identify what was supportive/missing in their journey, iii) practice listening skills and how to negotiate the support agreement between them and potential service users, iv) understand the scope of the peer supporter role, and v) learn to be a resource, including navigating a resource pack. |
| **WHAT MATERIALS AND PROCEDURES WERE USED?** | **Training Materials:**  Informational Resource packs  GDPR, Confidentiality and Scope of Practice  Referral pathway  Agreement documentation (between peer supporter and person being supported)  Evaluation form  **Procedures:**  Group work, brainstorming, skills practice (listening skills, agreement negotiation, use of resource pack) |
| **WHO PROVIDED THE INTERVENTION?** | Marie Keating Foundation, Oncology Psychologist, Health and Wellbeing Division, Health Service Executive |
| **HOW AND WHERE DELIVERED THE INTERVENTION?** | In a group setting, in-person and online. |
| **WHEN AND HOW MUCH?** | Two days consecutive face-to-face training and one half-day (online). Training to be supplemented by group check-in meetings quarterly (face-to-face or online), ongoing telephone support and WhatsApp contact. |
| **TAILORING AND MODIFICATIONS** | Changes made the process of peer supporter self-disclosure based on feedback and discussion. Guidance re: appropriate self-disclosure provided. |
| **HOW WELL PLANNED?** | Gaps in time between training and commencing peer supporter role should be avoided. In-person training/continuous professional development should be considered as preferred by peer supporters for discussion and support, with the option of informal peer-to-peer support outside formal sessions. |

Adopted from: Hoffmann, T.C., Glasziou, P.P., Boutron, I., Milne, R., Perera, R., Moher, D., et al. (2014) ‘Better reporting of interventions: Template for Intervention Description and Replication (TIDieR) checklist and guide’, BMJ, 348, g1687. doi:10.1136/bmj.g1687. <https://www.equator-network.org/reporting-guidelines/tidier/>
